# Drug-Induced Liver Injury After COVID-19 Vaccine

**DOI:** 10.7759/cureus.16491

**Published:** 2021-07-19

**Authors:** Rupinder Mann, Sommer Sekhon, Sandeep Sekhon

**Affiliations:** 1 Internal Medicine, Saint Agnes Medical Center, Fresno, USA; 2 Biology, Clovis North High School, Fresno, USA; 3 Gastroenterology and Hepatology, Saint Agnes Medical Center, Fresno, USA

**Keywords:** covid-19 vaccine, liver dysfunctions, covid-19 pandemic, covid-19, drug-induced liver injury

## Abstract

The first case of coronavirus disease 2019 (COVID-19) was reported in December 2019 in China. World Health Organization declared it a pandemic on March 11, 2020. It has caused significant morbidity and mortality worldwide. Persistent symptoms and serious complications are being reported in patients who survived COVID-19 infection, but long-term sequelae are still unknown. Several vaccines against COVID-19 have been approved for emergency use around the globe. These vaccines have excellent safety profiles with few reported side effects. Drug-induced hepatotoxicity is mainly seen with different drugs or chemicals. There are only a few reported cases of hepatotoxicity with vaccines. We present a case of liver injury after administration of the vaccine against the COVID-19 infection.

## Introduction

A novel coronavirus, severe acute respiratory syndrome coronavirus 2 (SARS-COV-2) causing coronavirus disease 2019 (COVID-19) emerged in December 2019 in Wuhan, China, resulting in an ongoing pandemic [1]. To date, it has caused more than 173 million cases and over 3.7 million death worldwide as per World Health Organization [2]. Although the respiratory system is the most common system affected by this disease, it affects multiple organ manifestations [3]. Despite international efforts to develop treatments for this disease, there are still limited therapeutic options available with remdesivir as the only Food and Drug Administration-approved drug [4]. Given the rapid spread, high morbidity, and mortality worldwide, a coordinated effort led to developing the vaccine in a year of first diagnosed case. Multiple COVID vaccines have been developed at an unprecedented rate. These vaccines have excelled safety and efficacy profiles [5-7]. The most common adverse effects reported with these vaccines included mild effects like pain at the vaccine site, fever, fatigue, headache, arthralgia, myalgia, lymphadenopathy, and severe effects like anaphylactic reaction [8]. Drug-induced hepatotoxicity is a common adverse event seen with prescription and nonprescription drugs [9]. There are few reported hepatotoxicity cases due to vaccines, namely anti-rabies vaccination-induced hepatotoxicity and autoimmune hepatitis due to influenza virus and hepatitis A and B vaccines [10-17]. We report a case of liver injury after receiving the COVID vaccine.

## Case presentation

A 61-year-old female with a known history of irritable bowel disease and cholecystectomy presented to the emergency department with generalized weakness, body aches, dry heaving, and a low-grade temperature of 99.9 Fahrenheit. The patient received a second dose of the Pfizer COVID-19 vaccine nine days before the start of symptoms. She was noted to have conjunctival icterus, mild generalized abdominal tenderness without guarding, or rigidity on physical examination. On admission, the patient's vitals were stable except for tachycardia with a heart rate between 90 and 110 beats/min.

**Figure 1:**
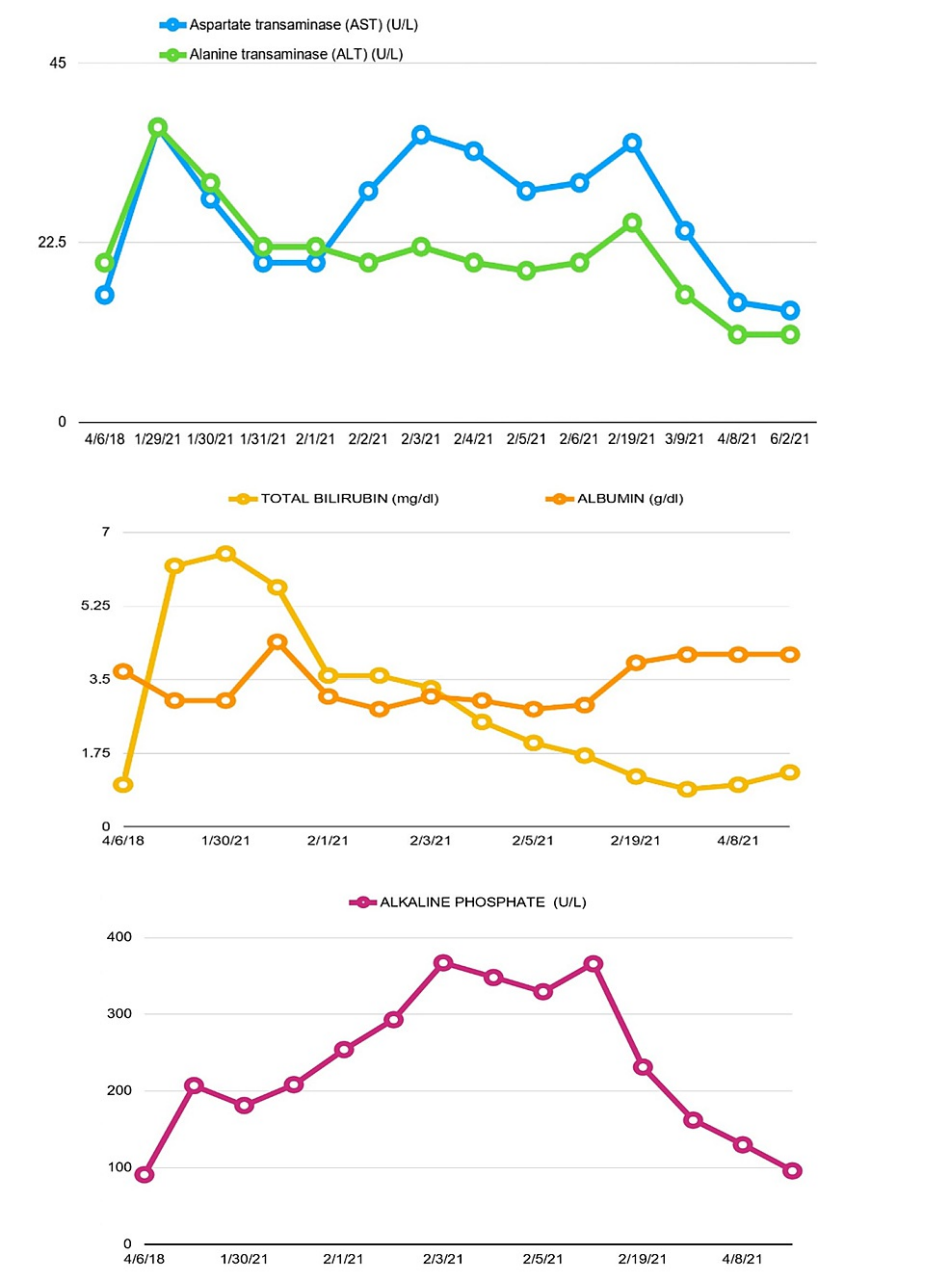
Graphs showing liver function test trends

Laboratory analysis was remarkable for elevated alkaline phosphatase (ALP) of 207 U/L, total bilirubin of 6.2 mg/dL, direct bilirubin of 3.9 mg/dL, white blood cell (WBC) count of 17.2 x 10^9^/L, and mildly elevated aspartate transaminase of 37 U/L (Table 1 and Figure 1). Abdominal ultrasound showed increased echogenicity within the liver compatible with fatty infiltrates, and common duct diameter was measured to be 6 mm. At the same time, CT of the abdomen with contrast showed no acute abnormalities. The patient was admitted to the hospital and started on empiric antibiotics for presumed cholangitis. Gastroenterology consultation was obtained. Magnetic resonance cholangiopancreatography without contrast showed no filling defect within the biliary duct, status post cholecystectomy, bile duct diameter within a normal range, and unremarkable liver. The patient remained afebrile, WBC trended down, and abdominal pain improved over the course of the hospital stay. Given these findings, infectious disease specialist recommended discontinuing antibiotics. Antibodies to liver/kidney microsomal type 1, smooth muscle, anti-mitochondrial, alpha-1 antitrypsin came back negative, and, additionally, ceruloplasmin, antinuclear antibody, alpha-fetoprotein, and viral serologies for hepatitis A, B, and C came back negative (Table 2). Liver biopsy showed minimal pallor suggesting slight edema along with scattered inflammatory cells consisting of small lymphocytes, scattered polymorphonuclear leukocytes, and few eosinophils, no evidence of florid duct lesion on interface hepatitis, and no evidence of fibrosis on trichrome and reticulin stain. 

**Table 1:** Liver function tests trend

Date	Aspartate transaminase (U/L)	Alanine transaminase (U/L)	Total bilirubin (mg/dL)	Alkaline phosphate (U/L)	Albumin (g/dL)
4/6/2018	16	20	1	91	3.7
1/29/2021	37	37	6.2	207	3
1/30/2021	28	30	6.5	181	3
1/31/2021	20	22	5.7	208	4.4
2/1/2021	20	22	3.6	254	3.1
2/2/2021	29	20	3.6	293	2.8
2/3/2021	36	22	3.3	367	3.1
2/4/2021	34	20	2.5	348	3
2/5/2021	29	19	2	329	2.8
2/6/2021	30	20	1.7	366	2.9
2/19/2021	35	25	1.2	231	3.9
3/9/2021	24	16	0.9	162	4.1
4/8/2021	15	11	1	130	4.1
6/2/2021	14	11	1.3	96	4.1

**Table 2:** Immunologic and infectious work-up for liver disease

Test	Results
Gamma-glutamyl transpeptidase	103 U/L (1-24 U/L reference range)
Hepatitis A IgM antibody	Negative
Hepatitis B surface antigen	Negative
Hepatitis B core IgM antibody	Negative
Hepatitis C antibody	Negative
Anti-liver/kidney microsomal antibody	Negative (≤20 = negative, reference range)
Ferritin	975.2 ng/mL (10.0-291.0 reference range)
Antinuclear antibody reflex	Negative
Smooth muscle antibody	Negative
Anti-mitochondrial antibody	Negative (≤20 = negative, reference range)
Ceruloplasmin	38 mg/dl (18-53 mg/dL reference range)

Given that all work-up for infection, autoimmune diseases, and any obstruction came back negative, the patient's clinical picture and laboratory findings were attributed as a liver injury due to the COVID-19 vaccine. Her liver function levels continued to trend down, and she was discharged from the hospital after a week of hospitalization. On the patient's follow-up with a gastroenterologist, abdominal pain was resolved, and her liver function test values normalized (Table 1 and Figure 1).

## Discussion

Drug-induced hepatotoxicity leads to nearly 10% of all cases of acute hepatitis and more than 50% cases of liver failure [18]. It is one of the common reasons for the withdrawal of medications from the market and modification of use [19]. It can be either type A (predictable), dose-related and short latent period in days, or type B (idiosyncratic), dose-independent, unpredictable, and variable latency [20,21]. Based on population-based studies, drug-induced liver injury incidence varies between 13.9 and 19.1 cases per 100,000 people per year [22,23]. Patients have either hepatocellular injury (three times upper limit of transaminase in comparison to ALP), cholestatic injury (three times increase in ALP comparison to transaminase), or mixed pattern (where both ALP and aminotransferase are three times upper limit) [24-26]. Most patients improve spontaneously after the removal of the offending drug. If acute liver failure (ALF) is suspected, early liver transplant referral is important due to high ALF mortality [25,27]. From the spontaneous reports from patients who received Pfizer/BioNTech BNT162b2 mRNA in the UK between 9/12/20 and 26/05/2021, there are reports of 45 patients having abnormal liver function analysis and three patients having drug-induced liver injury [28].

In this case, the review of medications and history did not reveal any other reason for hepatotoxicity. She also denied the use of any over-the-counter medications or supplements. Although it is rare with vaccination, the COVID-19 vaccine is likely the cause of hepatotoxicity in our patient based on a diagnosis of exclusion. In this case, the patient had a cholestatic pattern with elevated ALP and bilirubin with mild elevation in the transaminases.

Pfizer/BioNTech BNT162b2 mRNA trial included only 0.6% (217/37,706) patients with liver disease. Among patients with liver disease, 214 were with mild liver disease and only three with moderate to severe liver disease. This patient has underlying fatty liver disease. It is unclear if that was a likely risk factor for hepatotoxicity in this case [5]. Although only a small number were included in trials for Pfizer/BioNTech BNT162b2 mRNA, Moderna mRNA-1273, and the AstraZeneca/University of Oxford ChAdOx1-nCoV-19 chimpanzee adenovirus vector vaccine, both the American Association for Study of Liver Diseases and European Association for the Study of Liver recommend vaccination against SARS-COV-2 with these highly effective and safe vaccines, given a greater risk of health consequences from SARS-COV-2 infection in these patients [29,30].

Hepatotoxicity can occur with vaccines, even though it is more common with prescription and nonprescription drugs. So, the clinician should be watchful in patients showing clinical signs and symptoms after a vaccine.

## Conclusions

In summary, we presented a case of liver injury after the COVID-19 vaccine. We attributed the cause of liver injury to the COVID-19 vaccine, given no other cause in our patient after extensive work-up. There are reports of drug-induced liver injury and abnormal liver function analysis from the spontaneous reports from patients who received Pfizer/BioNTech BNT162b2 mRNA COVID-19 vaccine in the UK. The purpose of this manuscript is to raise awareness of potential side effects; it should not alter the recommendation of healthcare providers regarding vaccinations.
